# The Function of Sialidase Revealed by Sialidase Activity Imaging Probe

**DOI:** 10.3390/ijms22063187

**Published:** 2021-03-20

**Authors:** Akira Minami, Yuuki Kurebayashi, Tadanobu Takahashi, Tadamune Otsubo, Kiyoshi Ikeda, Takashi Suzuki

**Affiliations:** 1 Department of Biochemistry, School of Pharmaceutical Sciences, University of Shizuoka, Shizuoka 422-8526, Japan; kurebayashi@u-shizuoka-ken.ac.jp (Y.K.); takahasi@u-shizuoka-ken.ac.jp (T.T.); suzukit@u-shizuoka-ken.ac (T.S.); 2 Department of Organic Chemistry, School of Pharmaceutical Sciences, Hiroshima International University, Hiroshima 737-0112, Japan; t-ootubo@ps.hirokoku-u.ac.jp (T.O.); ikeda@hirokoku-u.ac.jp (K.I.)

**Keywords:** sialidase, BTP3-Neu5Ac, BTP9-Neu5Ac, hippocampus, memory, glutamate, pancreas, diabetes, skin, elastin, virus

## Abstract

Sialidase cleaves sialic acid residues from glycans such as glycoproteins and glycolipids. In the brain, desorption of the sialic acid by sialidase is essential for synaptic plasticity, learning and memory and synaptic transmission. BTP3-Neu5Ac has been developed for sensitive imaging of sialidase enzyme activity in mammalian tissues. Sialidase activity in the rat hippocampus detected with BTP3-Neu5Ac increases rapidly by neuronal depolarization. It is presumed that an increased sialidase activity in conjunction with neural excitation is involved in the formation of the neural circuit for memory. Since sialidase inhibits the exocytosis of the excitatory neurotransmitter glutamate, the increased sialidase activity by neural excitation might play a role in the negative feedback mechanism against the glutamate release. Mammalian tissues other than the brain have also been stained with BTP3-Neu5Ac. On the basis of information on the sialidase activity imaging in the pancreas, it was found that sialidase inhibitor can be used as an anti-diabetic drug that can avoid hypoglycemia, a serious side effect of insulin secretagogues. In this review, we discuss the role of sialidase in the brain as well as in the pancreas and skin, as revealed by using a sialidase activity imaging probe. We also present the detection of influenza virus with BTP3-Neu5Ac and modification of BTP3-Neu5Ac.

## 1. Introduction

The functional analysis of glycans has lagged behind that of nucleic acids and proteins. This is due to the extreme diversity of glycan structures, as well as the lack of tools to analyze the function of glycans. Recently, new tools have been developed to analyze the function of glycans. These tools enable us to understand the role of glycans from a different perspective. Sialidase is a hydrolytic enzyme that releases the non-reducing terminal sialic acid residues from sialoglycans. Sialic acid is abundant in the brain and is involved in various functions such as axonal outgrowth, neurotransmission, memory, and activities of voltage-gated sodium channel, voltage-gated calcium channels, *N*-methyl-D-aspartate (NMDA) receptor, and amino-3-hydroxy-5-methyl-4-isoxazolepropionate (AMPA) receptor [1,2,3]. Polysialic acid (PSA) is a linear homopolymer of sialic acid and can be detected in a wide range of eukaryotes, including vertebrates and invertebrates, and prokaryotes as well [4]. In the mammalian brain, PSAs are involved in the neural cell adhesion molecule (NCAM)-mediated inhibition of synaptogenesis and capture of brain-derived neurotrophic factor (BDNF) and dopamine [5,6]. Thus, the desorption of sialic acid by sialidase also plays an essential role in the regulation of brain function. In this review, we discuss the function of sialidase in the brain as well as in the pancreas and skin, as revealed by sialidase activity imaging probes. We will also present the detection of influenza viruses using sialidase activity imaging probes and the improvement of these probes.

## 2. Sialidase Activity Imaging Probes

Some artificial substrates for the detection of sialidase activity have been used to elucidate the function of sialidase. However, none of them were sufficient to visualize sialidase activity in tissues with high sensitivity. One of the most commonly used artificial substrates is 4-methylumbelliferyl-α-d-*N*-acetylneuraminic acid (4MU-Neu5Ac). 4MU-Neu5Ac is hydrolyzed by sialidase to release 4-methylumbelliferone (4MU), which emits fluorescence. Sialidase activity can be measured conveniently and with high sensitivity by measuring the fluorescence intensity of 4MU. However, 4MU has high diffusivity due to its high solubility in water, making it unsuitable for tissue staining. The chemiluminescent substrate NA-Star^®^ is capable of sensitively detecting the influenza virus with high sensitivity, but has a short luminescence half-life of about 5 min and is not suitable for tissue staining. PNP-Neu5Ac releases yellow-colored para-nitrophenol but has high diffusivity and is poorly distinguishable from the tissue color. The 5-bromo-4-chloroindol-3-yl-α-d-*N*-acetylneuraminic acid (X-Neu5Ac) is hydrolyzed by sialidase to release 5-bromo-4-chloro-3-hydroxyindole (X). The compound X is oxidized to produce an insoluble indigo pigment that stains the tissue. However, sialidase activity in mammals is weaker than that in the influenza virus and bacteria, and staining mammalian tissues with colorimetry of X-Neu5Ac has low sensitivity. Fast red violet (FRV) LB can be used as a reagent to fluoresce compound X. The imaging of sialidase activity in rat brains with high sensitivity is possible when X-Neu5Ac was used in combination with FRV LB [7,8]. However, the fluorescence is not completely attenuated to the background level by using sialidase inhibitors. The two-step process of hydrolysis of X-Neu5Ac by sialidase and subsequent fluorescence by FRV LB is presumably responsible for the low specificity of the staining.

## 3. Highly Sensitive Imaging Probe for Sialidase Activity

**Figure 1:**
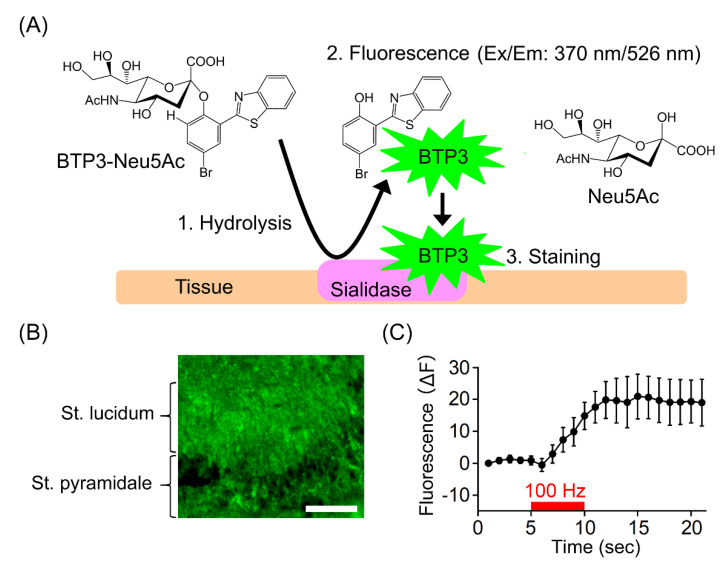
The rapid increase in sialidase activity in conjunction with neural activity. (**A**) Staining principles of BTP3-Neu5Ac. (**B**) Staining of rat hippocampal CA3 with BTP3-Neu5Ac. St, striatum. Scale, 0.1 mm. (**C**) Changes in sialidase activity at striatum lucidum by stimulation of the hippocampal mossy fibers (100 Hz, 5 s).

In order to compensate for the shortcomings of previous sialidase activity imaging, a high-sensitivity fluorescent probe for tissue staining has been developed, which can be stained in a single step by a hydrolysis reaction by sialidase [9]. 2-benzothiazol-2-yl-phenol (BTP) was used as a fluorescent dye for tissue staining. BTP is a bright fluorescent material with a large Stokes shift, low solubility in water and insensitivity to pH. The derivative of BTP exhibits various fluorescence wavelengths depending on the structure, allowing for a choice of suitable colors for staining. For commercial use, 2-(benzo[*d*]thiazol-2-yl)-4-bromophenol (BTP3) has been selected for its balance of visibility and ease of synthesis. A fluorescent probe, 2-(benzo[*d*]thiazol-2-yl)-4-bromophenyl-α-d-*N*-acetylneuraminic acid (BTP3-Neu5Ac), has been developed for imaging the enzymatic activity of sialidase [10,11]. The staining principle of BTP3-Neu5Ac is shown in Figure 1A. BTP3-Neu5Ac is a non-fluorescent compound with a structure consisting of BTP3 bound to sialic acid. BTP3-Neu5Ac releases BTP3 upon hydrolysis by sialidase (1) and emits a strong green fluorescence (2). As BTP3 is insoluble in water, it precipitates and stains the tissue (3). Staining is completed by placing an unfixed tissue section in a buffer containing BTP3-Neu5Ac (10–1000 μM) and incubating at room temperature for approximately 30 min, followed by washing the sections in buffer. In order to confirm the specificity of the staining, it is desirable to check that the fluorescence of the staining is abolished by the sialidase inhibitor.

There are four isozymes of mammalian sialidase, Neu1, Neu2, Neu3 and Neu4, which differ in their optimal pH, subcellular localization and substrate specificity. Under neutral conditions (pH 7.3), BTP3-Neu5Ac efficiently detects the enzymatic activity of Neu2 and Neu4. Under acidic conditions (pH 4.6), BTP3-Neu5Ac is able to detect the enzymatic activity of all sialidase isozymes, but is particularly efficient at detecting Neu1 and Neu3 [12]. Recently, imaging probes with higher staining resolution and artificial substrates that are stable under acidic conditions (pH 2.0) were developed [13,14].

## 4. Increase in Sialidase Activity in Conjunction with Neural Excitation

Staining rat brains with BTP3-Neu5Ac showed that the white matter regions such as the corpus callosum and the internal capsule have intense sialidase activity. In the hippocampus, relatively intense sialidase activity was detected in the stratum lucidum of CA3 regions projected by mossy fibers, which are the one of the major excitatory neurons of the hippocampus. (Figure 1B) [15]. When the mossy fibers were stimulated by long-term potentiation (LTP)-inducible high-frequency stimuli, the sialidase activity detected with BTP3-Neu5Ac on the surface of the plasma membrane in the stratum lucidum was increased 1–2 s after the onset of stimulation (Figure 1C). In addition to the LTP-inducible stimuli, sialidase activity was also increased by neuronal depolarization induced with a high concentration of potassium, by activation of NMDA receptors or kainic acid receptors, and by the administration of BDNF or LTP-inducing reagent forskolin. On the contrary, sialidase activity is attenuated by the inhibitory neurotransmitter GABA. Thus, sialidase activity rapidly changes enzymatic activity in response to the state of neural activity [15]. The increase in sialidase activity induced by glutamate occurs not only in the neurons but also in the glial cell astrocytes. Considering that depolarizing stimuli do not increase sialidase activity in the culture medium and do not increase the mRNA levels of any sialidase isozyme, it is presumed that the increase in sialidase activity by neural excitation is not due to increased sialidase secretion or sialidase expression. Therefore, the increase in sialidase activity on the cell surface may be due to changes in the subcellular localization of sialidase or factors that affect sialidase activity.

## 5. In Vivo Sialidase Activity Monitoring

**Figure 2:**
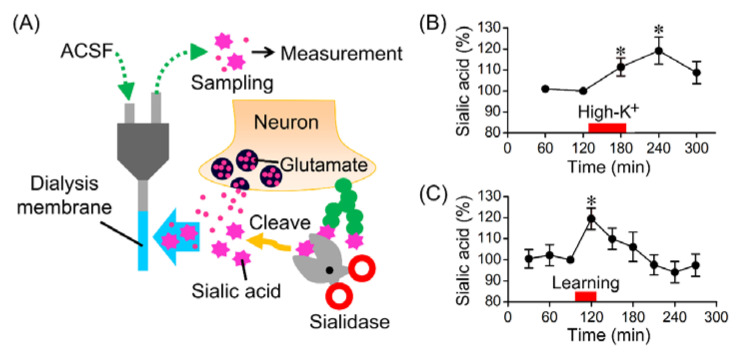
Sialic acid desorption during memory formation. (**A**) In vivo monitoring of sialic acid desorption using a microdialysis method. (**B**,**C**) Increase in the amount of sialic acid in the extracellular fluid of the rat hippocampus by depolarizing stimulation (**B**) or during context learning with fear conditioning (**C**) (* *p* < 0.05, vs. basal level).

Sialic acid desorption on the cell surface can be detected in in vivo conditions using the microdialysis method. As shown in Figure 2A, free sialic acids are collected from the extracellular fluid in the brain via a microdialysis membrane. Changes in the sialidase activity on the cell surface can be indirectly identified by quantifying the sialic acid in the perfusate. Indeed, the administration of exogenous sialidase into the rat hippocampus increases the amount of sialic acid collected from the extracellular fluid of the hippocampus via the microdialysis membranes. In contrast, the amount of sialic acid collected from the extracellular fluid is reduced when endogenous sialidase is inhibited with sialidase inhibitors. For sensitive determination of sialic acids, pre-column fluorescence labeling of sialic acids with 1,2-diamino-4,5-methylenedioxybenzene in high-performance liquid chromatography is frequently used. However, sialic acid cannot be quantified in the presence of 2,3-dehydro-2-deoxy-*N*-acetylneuraminic acid (DANA), which is a commonly used sialidase inhibitor, because the fluorescently labeled compound of sialic acid and DANA would be the same substance. Thus, when sialidase inhibitors are used in experiments to monitor sialidase activity by using microdialysis, sialidase inhibitors with a different structure from DANA, such as 2,3-dehydro-2-deoxy-*N*-propanoylneuraminic acid (DPNA) and 2,3-dehydro-2-deoxy-*N*-glycolylneuraminic acid (DGNA), are used.

Sialic acid desorption from glycans in conjunction with neural excitation can be detected by using the in vivo monitoring of sialidase activity [15]. The amount of free sialic acid collected from the extracellular fluid of the hippocampus was increased by depolarization induced by high concentrations of potassium (Figure 2B). Sialic acid desorption in hippocampus after neural excitation can be also confirmed by lectin staining. The amount of sialic acid residue at the non-reducing terminal detected by MAA lectin (for recognition of Neu5Acα2-3Gal-β(1–3)-GalNAc structure) was reduced after high-potassium treatment. In contrast, the amount of galactose residue at the non-reducing terminal detected by PNA lectin (for recognition of Gal-β(1–3)-GalNAc structure) was increased after high-potassium treatment. Thus, the increase in sialidase activity after neural excitation is considered to be sufficient to cleave the sialic acids from glycans.

## 6. Role of Sialidase in Memory Formation

Sialidase is essential for hippocampus-dependent memory formation. Hippocampal-dependent spatial memory in rats assessed in the Morris water maze is attenuated by administration of DANA or Neu4 siRNA into the hippocampus [12]. DANA also attenuates the LTP at synapses between the hippocampal mossy fibers and CA3 pyramidal cells, and between Schaffer collaterals and CA1 pyramidal cells [12,16]. Contextual learning with fear conditioning increased the amount of sialic acid released into the hippocampal extracellular fluid (Figure 2C). These findings indicate that sialic acid desorption by sialidase during contextual learning is essential for memory formation.

PSAs expressed in the mossy fiber terminals are involved in synaptogenesis. Mossy fibers are replaced by new neurons approximately every three months and regularly form new synapses. Immature mossy fiber terminals express PSAs. In the PSA-expressing mossy fiber terminals, invagination from the CA3 pyramidal cell dendrites is observed during the formation of postsynaptic spines [17]. PSA expression is no longer observed in mature mossy fiber terminals. The desorption of sialic acid at the appropriate time and location is important for proper synaptogenesis in the mossy fiber terminals [6,17]. Homophilic binding of NCAM is inhibited by repulsion of the PSA due to its negative charge. An increase in sialidase activity in conjunction with neural excitation during memory formation might be involved in synaptic formation via sialic acid desorption from NCAM.

## 7. Role of Sialidase in the Glutamate Release

**Figure 3:**
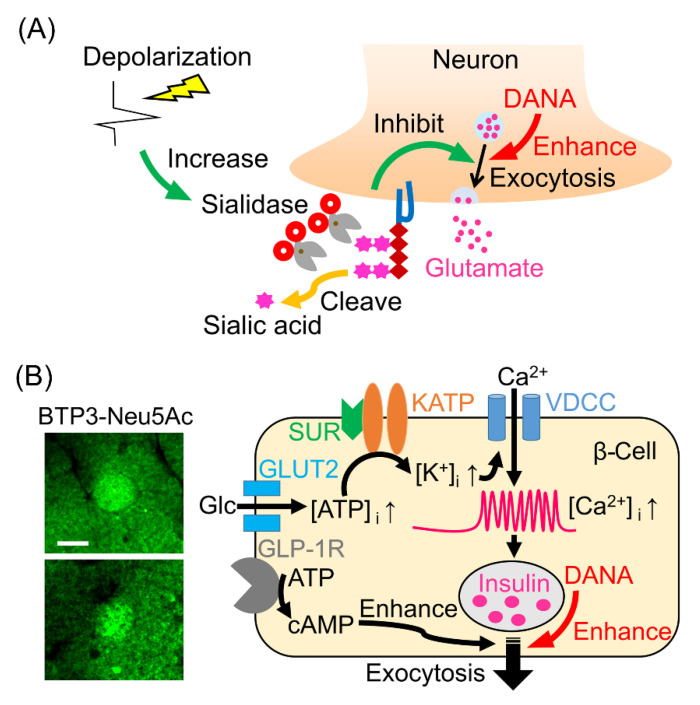
Effects of the sialidase inhibitor DANA on the release of glutamate and insulin. (**A**) Negative feedback on neural excitation by sialidase. DANA promotes the release of glutamate. (**B**) Distribution of sialidase activity in the mouse pancreas (left, scale, 0.1 mm). DANA promotes insulin secretion (right). Glc, glucose; GLP-1R, glucagon-like peptide-1 receptor; GLUT2, glucose transporter 2; KATP, ATP-sensitive potassium channel; SUR, sulfonylurea receptor; VDCC, voltage-dependent calcium channel.

The extracellular glutamate level in the rat hippocampal mossy fiber terminal regions measured by using microdialysis was increased with sialidase inhibitors. Synaptic vesicle exocytosis induced by high-potassium treatment was also enhanced with DANA in primary cultured hippocampal neurons. These findings show that sialidase suppresses glutamate release in hippocampal neurons [18]. An increase in sialidase activity in conjunction with neural excitation might play a role in the negative-feedback mechanisms for the release of the excitatory neurotransmitter glutamate (Figure 3A). Sialic acids bound to *N*-linked glycopeptides function as potential modulators of neurotransmitter release. It has been suggested that the changes in the amount of sialic acid modification after neuronal depolarization by sialidases and sialyltransferases affect the function of membrane glycoproteins [19]. Sialic acids bound to ganglioside GQ1b as well as *N*-linked glycopeptides promote the calcium signals and acetylcholine release. The sialidase inhibitor DANA increases the expression of ganglioside GQ1b/GT1a, resulting in enhancement of the intracellular calcium increase due to neuronal firing. Thus, the inhibition of glutamate release by sialidase involves the suppression of intracellular calcium influx via sialic acid desorption from the gangliosides.

## 8. Role of Sialidase in the Pancreatic Islets

Mammalian tissues other than the brain were also stained with BTP3-Neu5Ac. In the mouse pancreas, the mouse pancreatic islets showed relatively intense sialidase activity (Figure 3B left). The sialidase activity in the pancreatic islets is mainly mediated by Neu3. Neurotransmitter exocytosis and insulin secretion have many common mechanisms, including plasma membrane fusion and vesicle recycling processes. Therefore, DANA was thought to affect not only glutamate release but also insulin secretion. In fact, administration of DANA to mice promoted insulin secretion and lowered blood glucose levels (Figure 3B right). Interestingly, the promotion of insulin secretion did not occur under hypoglycemia after fasting. Hypoglycemia is a serious side effect of anti-diabetic drugs that promote insulin secretion. Sialidase inhibitors are expected to be used as anti-diabetic drugs to avoid the hypoglycemia side effect.

Sialidases are widely involved in the homeostasis of insulin-mediated glucose metabolism. Neu1 regulates insulin signaling for energy metabolism and glucose uptake without affecting insulin production [20]. Desialylation of the insulin receptor by Neu1 induces active conformation of the insulin receptor dimer [21]. Neu3 is associated with insulin sensitivity and glucose tolerance [22,23,24]. Neu3 overexpression in the liver improves insulin sensitivity and glucose tolerance [25]. Ganglioside GM3 induces insulin resistance [26] and is efficiently hydrolyzed by Neu3 [27,28]. Conversely, transgenic mice overexpressing Neu3 in the whole body developed severe insulin-resistant diabetes mellitus [23]. DANA improved insulin sensitivity and consequently alleviated the hyperglycemia induced by chronic intravenous injection of elastin-derived peptides [29]. It has also been reported that sialidase inhibitors may worsen insulin sensitivity. Our data show that acute or chronic administration of DANA does not affect insulin sensitivity [30]. Thus, the effect of sialidase on insulin sensitivity remains unclear. DANA inhibits all mammalian sialidase isozymes [31,32]. Sialidase isozyme-selective inhibitors have been developed for Neu1, Neu2, Neu3, and Neu4 [32,33,34,35]. In order to reduce the side effects caused by sialidase inhibition, sialidase isozyme selective inhibitors are considered to be useful in the treatment of diabetes.

## 9. Role of Sialidase in the Skin

The extracellular matrix components in the skin are mainly composed of collagen, glycosaminoglycans and elastin. Sialidase isozyme Neu1 has been reported to be involved in the elastic fiber assembly [36,37]. Neu1 constitutes an elastin receptor complex with elastin-binding protein (EBP), which has been identified as a splicing variant of β-galactosidase, and protective protein/cathepsin A on the cell surface. Cleavage of sialic acid from the glycoprotein microfibrils by Neu1 allows EBP to bind to the microfibrils. In the next step, tropoelastin, a precursor protein to elastin, is released from EBP, assembled around the microfibrils, and cross-linked between the molecules to form mature elastin fibers [37].

When the rat tissues, including skin, were stained with BTP3-Neu5Ac at pH 7.3, intense sialidase activity was detected in the dermis and muscle compared to that in adipose tissue [38]. At pH 7.3, BTP3-Neu5Ac is efficiently hydrolyzed by the sialidase isozymes Neu2 and Neu4 [12]. In fact, the expression of Neu2 mRNA in the dermis and muscle was abundant compared with that in adipose tissue. Neu2 is located mainly in the cytoplasm, but also in the plasma membrane [39]. Neu2 hydrolyzes glycoproteins, oligosaccharides, and gangliosides, and has an optimum pH of 6.0–6.5 [40]. Intense sialidase activity in the dermis and muscle at neutral pH was presumed to be due to Neu2.

Since Neu2 can act at neutral pH in the extracellular space, exogenous Neu2 delivered to the dermis for transdermal administration may efficiently release sialic acid from microfibrils in the extracellular space. Thus, the effect of Neu2 on elastin production was investigated by delivering exogenous Neu2 to the dermis. The stratum corneum constitutes a permeation barrier of the skin. An optimal selection of penetration enhancers is required for effective and noninvasive transdermal administration [41,42,43]. Choline and geranate (CAGE) is one of the deep eutectic solvents and has been reported to show efficient transdermal delivery of various types of drugs from low molecular weight compounds to high molecular weight compounds such as peptides and proteins [44,45,46]. The enzymatic activity of sialidase is maintained in CAGE solution and in rat skin after transdermal delivery of sialidase with CAGE. Transdermal delivery of Neu2 with CAGE increased the amount of elastin in the dermis [38]. Thus, it was suggested that not only Neu1 but also Neu2 is involved in elastin production.

## 10. Improvement of Aged Skin by Sialidase

Elastin fibers naturally decrease with intrinsic aging in the skin protected from sunlight [47,48]. Elastin reduction in the skin causes wrinkles, sagging, and skin disorders [49,50]. Since elastin turnover takes time, it is difficult to regenerate elastin once it is lost [51,52]. Changes in sialidase activity with aging were examined. Interestingly, sialidase activity measured using 4MU-Neu5Ac at pH 7.3 in rat dermis was found to decrease with aging [38]. Therefore, supplementation of sialidase by transdermal administration may restore the loss of elastic fibers associated with aging. The senescence-accelerated mouse (SAM) is characterized by an accelerated aging process, a short lifespan, and early onset and rapid progression of age-related pathological phenotypes similar to human senile diseases [53,54,55]. Several strains of SAM prone (SAMP) have been established at Kyoto University in Japan as mouse strains that show a rapid age-dependent increase in senescence scores from mouse colonies resulting from an unexpected cross between AKR/J mice and an unknown strain [54].

SAMP1 mice exhibit increases in elastic fibers and epidermal thickness that develop into overt elastosis, the characteristics of which are similar to those of human photoaging [56]. SAMP8 mice exhibit precocious skin aging similar to that of aged wild-type mice [57]. Transdermal administration of sialidase from *Arthrobacter ureafaciens* with CAGE increased the amount of elastin in the skin of SAMP1 and SAMP8. Rodent and porcine skin have been widely used to study skin permeability, as human skin varies widely in drug permeability depending on skin site, age and race. Among rodents, rat skin is the most structurally similar to human skin and is often used for in vivo pharmacokinetics, pharmacological and toxicological studies [58].

The lysosomal enzyme Neu1 has an optimum pH of 4.4–4.6 and shows a rapid decrease in enzyme activity after purification. Since Neu2 is stable after purification and works in an extracellular neutral pH environment, Neu2 is considered to be more appropriate for medical applications and commercial use. Elastin levels are reduced not only by skin aging but also by dermatochalasis [59], pseudoxanthoma elasticum [60], Williams–Beuren syndrome [61], and skin disorders due to obesity [62], which deteriorate the skin environment. Therefore, transdermal administration of sialidase is expected to be useful for the improvement of wrinkles and skin disorders caused by loss of elastin.

## 11. Detection of Influenza Viruses

Some viruses have viral surface proteins that exhibit intense sialidase activity. Influenza A and B viruses have viral neuraminidase glycoproteins (NAs) that exhibit high sialidase activity [63] and are expressed on the membranes of infected cells in the late post-infection period. Some reports have shown that sialidase substrates such as 4MU-Neu5Ac [64,65,66] and the 1,2-dioxetane derivative of Neu5Ac (NA-star) [67] can detect the sialidase activity of influenza viruses. In addition, BTP3-Neu5Ac enabled a cytochemical imaging assay for influenza virus sialidase activity in living cells [68]. Influenza virus-blotted membrane, influenza virus-infected cells, and influenza virus NA-expressing cells were clearly stained by BTP3-Neu5Ac. Fluorescence was completely inhibited in the presence of specific sialidase inhibitors of influenza A and B viruses. All tested influenza virus strains, including influenza A H1N1, H3N2 and B viruses, can be detected in infected cells by BTP3-Neu5Ac, suggesting that the BTP3-Neu5Ac assay is applicable to the sialidase activity of all influenza viruses.

The usefulness of BTP3-Neu5Ac for virus isolation has also been demonstrated. Influenza A and B viruses form focuses on the cell monolayer overlaid with agarose. Simply dropping the BTP3-Neu5Ac solution into the agarose-containing medium resulted in clear staining of the viral focuses. Influenza viruses isolated from the BTP3-stained focus retained their viral replication ability, confirming the usefulness of BTP3-Neu5Ac for virus isolation. Thus, BTP3-Neu5Ac would be a powerful tool not only for virus detection but also for virus isolation. The usefulness of BTP3-Neu5Ac for histochemical staining of virus-infected tissues in vivo has also been demonstrated. Lung sections from influenza virus-infected mice were clearly stained by BTP3-Neu5Ac. Since many viruses other than influenza viruses have sialidase activity, BTP3-Neu5Ac can detect many viruses other than influenza viruses, including human parainfluenza virus and mumps virus [69,70,71,72]. The use of sialidase inhibitors specific for influenza virus NA, such as zanamivir, makes it possible to detect influenza A and B viruses separately from other viruses.

Histochemical and cytochemical detection and RT-PCR have been conventionally used for the detection of influenza viruses. Cytochemical detection of virus infection provides important information for both laboratory research and hygiene survey. Such chemical detection has traditionally been performed by immunochemical methods that require specific antiviral antibodies after cell fixation. In contrast, BTP3-Neu5Ac does not require specific antiviral antibodies or cell fixation, and allows for easy, rapid, and highly sensitive detection. The RT-PCR method requires highly specific primers to detect target genes, which may make the detection of the occurrence of news of the subtype virus somewhat inconvenient. In addition, the RT-PCR method requires a more complex protocol compared to the BTP3-Neu5Ac assay. The BTP3-Neu5Ac assay is expected to be applicable to all influenza viruses with sialidase activity, regardless of subtype, host, or gene variability. Thus, the new assay has many advantages over traditional methods for the detection of viral infections.

Influenza virus-specific neuraminidase inhibitors (NAIs) are commonly used in the clinical treatment of influenza. However, some influenza A and B viruses resistant to NAIs have emerged in nature. We also performed new assays to selectively detect and rapidly and conveniently isolate NAI-resistant viruses by live fluorescence imaging of viral sialidase activity. NAI-resistant viruses maintain their sialidase activity even in the presence of NAIs. Infected cells and focuses (infected cell populations) of an NAI-resistant virus were selectively stained by BTP3-Neu5Ac in the presence of NA, resulting in high-efficiency isolation of the resistant viruses [73].

By using a combination of a virus-concentrated membrane in a centrifugal filter unit and BTP3-Neu5Ac, the sialidase activity of influenza neuraminidase can be easily detected on the membrane by green fluorescence under UV irradiation with a handheld UV flashlight [74]. The assay can be completed within 15 min. The detection sensitivity was shown to be equal to or higher than the sensitivities of commercial immunochromatographic kits. Fluorescence staining of NAI-resistant sialidase activity will be a powerful method for the study of the NAI resistance mechanism, for public monitoring of NAI-resistant viruses, and for the development of a new NAI that shows an effect on various NAI-resistant mutations.

## 12. Modification of BTP3-Neu5Ac

BTP3-Neu5Ac is a powerful tool for detecting sialidase activity in viruses and infected cells. Recently, an attempt was made to modify BTP3-Neu5Ac. Probably due to the diffusivity of BTP3 itself and intense sialidase activity of influenza viruses compared to mammals, a slight diffusion of BTP3 fluorescence from influenza virus-infected cells into adjacent non-infected cells was observed as the reaction time of in situ imaging became longer. Therefore, an attempt was made to enhance the performance of in situ imaging with BTP3-Neu5Ac by introducing a linear hydrocarbon chain into BTP to increase its hydrophobicity [14]. The derivative with linear hydrocarbon chains showed significantly lower fluorescence diffusivity after the sialidase reaction compared to BTP3-Neu5Ac. In addition, the degree of unsaturation and introduced position of the hydrocarbon chain in the BTP affected the fluorescence brightness and intensity. The fluorescence intensity was stronger for aklynyl-BTP than for alkenyl- or aklyl-BTP, and at the *m*-position it was stronger than at the *p*-position. The chain length of the alkyne introduced into BTP also affected the fluorescence staining. BTP-Neu5Ac, in which 1-nonyne was introduced into the structure of BTP, showed less diffusivity after staining of influenza virus-infected cells than the compound with 1-pentyne. BTP-Neu5Ac with 1-nonyne at the *m*-position of BTP, 2-(benzo[*d*]thiazol-2-yl)-5-(non-1-yn-1-yl)phenyl-α-D-*N*-acetylneuraminic acid (BTP9-Neu5Ac), showed the least diffusible fluorescence imaging of influenza virus-infected cells.

Probably because of its improved membrane permeability, the modified BTP-Neu5Ac enabled imaging of intracellular sialidase activity in live influenza virus-infected cells. When the NA-induced extracellular sialidase activity was inhibited by zanamivir, BTP3-Neu5Ac hardly stained influenza virus-infected cells. On the other hand, BTP-Neu5Ac with 1-nonyne at the m-position stained zanamivir-treated cells brilliantly. Intracellular NA and its sialidase activity in influenza virus-infected cells were stained using anti-NA antibody and the modified BTP-Neu5Ac, respectively. The fluorescence image obtained by the modified BTP-Neu5Ac was almost consistent with the immunostaining image obtained by the anti-NA antibody. Some reports suggest that NA is involved in virus morphogenesis and budding [75]. Enzymatic activity-dependent imaging of intracellular NA with sialidase imaging probes makes it possible to analyze how sialidase activity of NA works in cells, and may reveal new functions of NA in influenza virus infection. The newly developed BTP9-Neu5Ac is expected to be a useful tool to contribute to the progress of sialidase research in various fields.

## 13. Concluding Remarks

The structure of cell surface glycan had been thought to change over a relatively long timeframe (days or hours) due to structural modifications within the cell. However, using BTP3-Neu5Ac, it has been shown that the enzymatic activity of sialidase in the hippocampus increases in a short timeframe (in seconds) in conjunction with neuronal excitation. BTP3-Neu5Ac is expanding its range of use, not only for the investigation of physiological functions but also for the detection of colorectal cancer and the influenza virus [9,74]. BTP derivatives can also be used as substrates for hydrolases other than sialidases, such as galactosidase [76]. The knowledge of the enzyme activity distribution of various glycan hydrolase in the tissues is expected to contribute to the elucidation of the glycan functions.

## Abbreviations

AMPA	amino-3-hydroxy-5-methyl-4-isoxazolepropionate
BDNF	brain-derived neurotrophic factor
BTP	2-(benzo[*d*]thiazol-2-yl)-4-bromophenol
BTP3-Neu5Ac	2-(benzo[*d*]thiazol-2-yl)-4-bromophenyl-α-d-*N*-acetylneuraminic acid
BTP9-Neu5Ac	2-(benzo[*d*]thiazol-2-yl)-5-(non-1-yn-1-yl)phenyl-α-d-*N*-acetylneuraminic acid
CAGE	choline and geranate
DANA	2:3-dehydro-2-deoxy-*N*-acetylneuraminic acid
DGNA	2,3-dehydro-2-deoxy-*N*-glycolylneuraminic acid
DPNA	2,3-dehydro-2-deoxy-*N*-propanoylneuraminic acid
EBP	elastin-binding protein
FRV	fast red violet
LTP	long-term potentiation
NA	viral neuraminidase glycoprotein
NAI	influenza virus-specific neuraminidase inhibitor
NCAM	neural cell adhesion molecule
NMDA	*N*-methyl-D-aspartate
PSA	polysialic acid
SAM	senescence-accelerated mouse
SAMP	senescence-accelerated mouse prone
X	5-bromo-4-chloro-3-hydroxyindole
X-Neu5Ac	5-bromo-4-chloro-3-hydroxyindole derivative
4MU	4-methylumbelliferone
4MU-Neu5Ac	4-methylumbelliferyl-α-d-*N*-acetylneuraminic acid
